# Wip1 is associated with tumorigenity and metastasis through MMP-2 in human intrahepatic cholangiocarcinoma

**DOI:** 10.18632/oncotarget.18074

**Published:** 2017-05-23

**Authors:** Sulai Liu, Bo Jiang, Hao Li, Zili He, Pin Lv, Chuang Peng, Yonggang Wang, Wei Cheng, Zhengquan Xu, Wei Chen, Zhengkai Liu, Bao Zhang, Shengqian Shen, Shuanglin Xiang

**Affiliations:** ^1^ Department of Hepatobiliary Surgery/Hunan Research Center of Biliary Disease, Hunan Provincial People's Hospital/The First Affiliated Hospital of Hunan Normal University, Changsha, Hunan Province, People's Republic of China; ^2^ Department of Orthopaedics, The First Affiliated Hospital of Fujian Medical University, Fuzhou, Fujian Province, People's Republic of China; ^3^ Department of Thoracic, The First Affiliated Hospital of Anhui Medical University, Hefei, Anhui Province, People's Republic of China; ^4^ Key Laboratory of Protein Chemistry and Developmental Biology of State Education Ministry of China, College of Life Science, Hunan Normal University, Changsha, Hunan Province, People's Republic of China

**Keywords:** intrahepatic cholangiocarcinoma, Wip1, MMP-2, prognosis

## Abstract

Wip1 has been shown to correlate with the metastasis/invasion of several tumors. This study was designed to investigate the clinical significance and biological function of Wip1 in intrahepatic cholangiocarcinoma (ICC). The expression of Wip1 was investigated in sixty human ICC biopsy samples by immunohistochemistry. Transient and stable knockdown of Wip1 in two human ICC cells (ICC-9810 and SSP25) were established using short hairpin RNA expression vector. Immunohistochemistry revealed that Wip1 was up-regulated in human ICC tissues (47/60, 78.3%). High levels of Wip1 in human ICC correlated with metastasis to the lymph metastasis (P=0.022). Genetic depletion of Wip1 in ICC cells resulted in significantly inhibited proliferation and invasion compared with controls. Most importantly, Wip1 down-regulation impaired tumor migration capacity of ICC cells *in vivo*. Subsequent investigations revealed that matrix metalloproteinase-2 (MMP-2) is an important target of Wip1. Consistently, in human ICC tissues, Wip1 level was positively correlated with MMP-2 expression. Taken together, our founding indicates that Wip1 may be a crucial regulator in the tumorigenicity and invasion of human ICC, Wip1 exerts its pro-invasion function at least in part through the MMP-2 signaling pathway, suggesting Wip1 as a potential therapeutic target for ICC.

## INTRODUCTION

Intrahepatic cholangiocarcinoma (ICC) is an aggressive malignancy with poor prognosis that makes up 10-25% of all primary hepatic cancers. However, its mortality and morbidity are increasing in recent years [1, 2]. On the other hand, ICC arises from the intrahepatic biliary tree and continues to be characterized by a poor prognosis, less than 20% to 50% of the patients alive 5 years after curative resection, and a high recurrence/metastasis rate [2, 3]. Radical surgical resection remains the first-line approach and presents most effective therapy of cure in the treatment of ICC [4];however, most patients have advanced disease at the time of diagnosis and are not suitable candidates for surgery [5, 6]. Therefore, specific and new biomarkers that can indicate the risk of morbidity and recurrence are clearly necessary for the development of novel therapeutic options for patients with ICC.

Wip1 (wild-type p53-induced phosphatase 1) belongs to PP2C family and is encoded by PPM1D gene, which inhibits p53 functions and activity in cancer cells. Wip1 is emerging as an important oncogene by virtue of its negative control on several key cancer suppressor pathways, including P38-MARK, Dkk3-Wnt, DNA damage response (DDR) [7–11]. Wip1 dephosphorylates many proteins, including ataxia-telangiectasia mutated (ATM), p53, p38, Mdm2, Mdm4, Chk1, Chk2, and UNG2 [12, 13] [7]. These proteins that belong DDR/checkpoint markers, are often decreased in DNA damage response pathways, which contribute to sensing and repairing DNA damage. Others and we previously studies showed that Wip1 has been subsequently found amplified and more recently mutated in a significant fraction of human cancers including kidney [14], breast [15], lung [16], ovarian [17], neuroblastoma [18]. Although the abnormalities of Wip1 have been efficiently investigated in some kinds of tumors, there has been no study evaluating the relationship between expression of Wip1 in ICC cells and patients’ tissues. In this study, we reported Wip1 expression occurs in ICC tissue specimens, and determined its relationship with the expression of AFP, GGT, Ki67, CA199, P53 and clinicopathological characteristics. Furthermore, we investigated the roles of Wip1 expression in biologic behavior of ICC cells.

## RESULTS

### The expression of Wip1 was elevated in ICC tissues and associated with hepatic hilar lymph nodes metastasis

**Figure 1:**
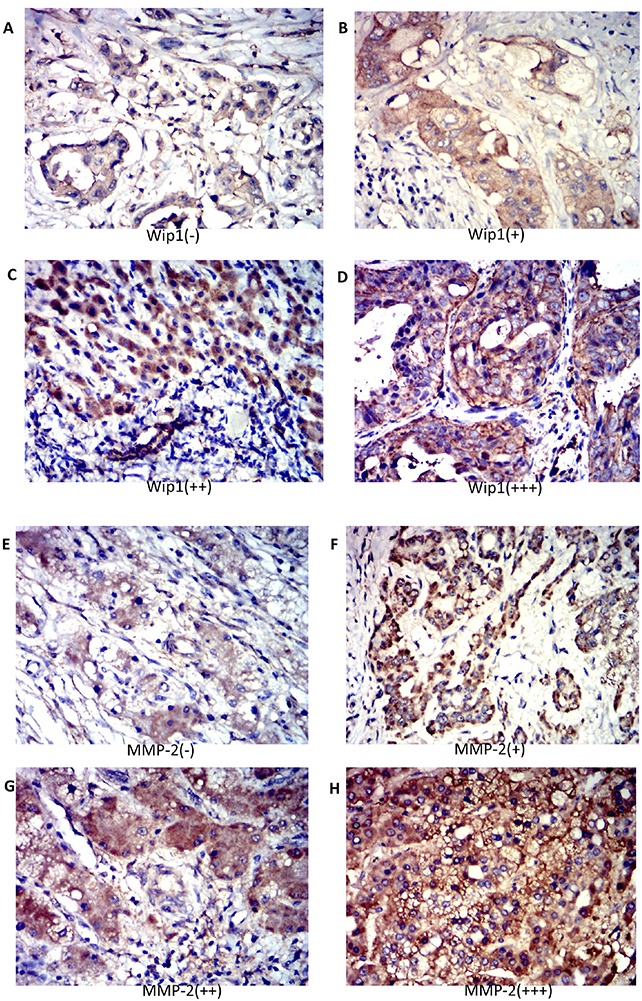
Expression of Wip1 and MMP-2 in human ICC tissues Immunohistochemistry revealed no staining (-) **(A)** low staining (+) **(B)** moderate staining (++) **(C)** and strong staining (+++) **(D)** for Wip1 and MMP-2 in human ICC tissue. The Wip1 level was positively correlated with MMP-2 expression in human ICC tissue **(A-H).** Magnification: 200×;*P<0.05.

To assess the Wip1 protein expression in ICC species, immunostaining was strictly performed according our previous method [14, 19] in 60 pairs of human ICC and normal tissue samples. Immunohistochemistry data showed that Wip1 expression was significantly up-regulated in ICC tissues compared with the normal samples (Figure 1). Wip1 protein was shown mainly in the cytoplasm and membrane of ICC cells, while our data showed Wip1 protein were rare detectable in normal tissues (Figure 1A). In general, high Wip1 expression (++ or +++) was observed in 47 of 60 tumor samples (78.3%; Figure 1C,1D), whereas low Wip1 expression (- or +) was noted in 13 of 60 tumor samples (21.7%; Figure 1B).

The correlations between Wip1 protein expression and clinicopathological characteristics of ICC were analyzed in Table 1. The level of Wip1 expression in ICC tissues was significantly correlated with nerve infiltration (P=0.035) and lymphatic metastasis (P<0.001). Furthermore, there were no significant differences observed between Wip1 expression and other clinicopathologic features, such as gender (P=0.79), age (P=0.86), Tumor differentiation (P=0.82), HBV infection (P=0.34), Tumor focality(P=0.75), Clinical T stage (P=0.32) or Vascular invasion (P=0.33).

**Table 1:** Correlation between Wip1 expression and 60 patients with ICC

Variables		Wip1		
		Positive (n=47)	Negative (n-13)	*P*
Sex	Male	27	8	*0.79*
	Female	20	5	
Age	≤60	12	3	*0.86*
	>60	35	10	
Tumor differentiation	Well	20	6	*0.82*
	Poor	27	7	
Nerve infiltration	Yes	33	5	***0.035***
	No	14	8	
Lymphatic metastasis	Yes	35	3	***<0.001***
	No	12	10	
HBV infection	Yes	32	7	*0.34*
	No	15	6	
Tumor focality	Solitary	42	12	*0.75*
	Multiple	5	1	
Vascular invasion	Yes	21	7	*0.32*
	No	26	6	
Clinical T stage	T1-2	18	4	*0.33*
	T3-4	29	9	

### Wip1 expression was correlated with P53, CA-199 and MMP-2 levels in ICC cases

The association between Wip1 expression and the expression of P53, CA-199, AFP, Ki67, GGT and MMP-2 in ICC cases were shown in Table 2. Our data show that the Wip1 protein expression was significantly associated with the P53/CA-199/MMP-2 (P=0.014, P=0.016 and P=0.008, respectively, Table 1). There were no correlation between the expression of Wip1 and any other of immunohistochemical findings, including AFP, Ki67 and GGT in ICC tissues.

**Table 2:** Association between the expression of wip1 and other markers in ICC

Variables	Wip1 expression		
	Positive(n=47)	Negative(n=13)	*P*
CA-199			***0.015***
Positive	40	7	
Negative	7	6	
AFP expression			
Positive	24	8	*0.5*
Negative	23	5	
P53 expression			***0.014***
Positive	18	10	
Negative	29	3	
Ki67 expression			*0.86*
Positive	35	10	
Negative	12	3	
GGT expression			*0.59*
Positive	22	5	
Negative	25	8	
MMP-2 expression			***0.008***
Positive	41	7	
Negative	6	6	

### Transfection of Wip1-shRNA repressed the proliferation and invasion capacity of ICC-9810 and SSP25 cells

**Figure 2:**
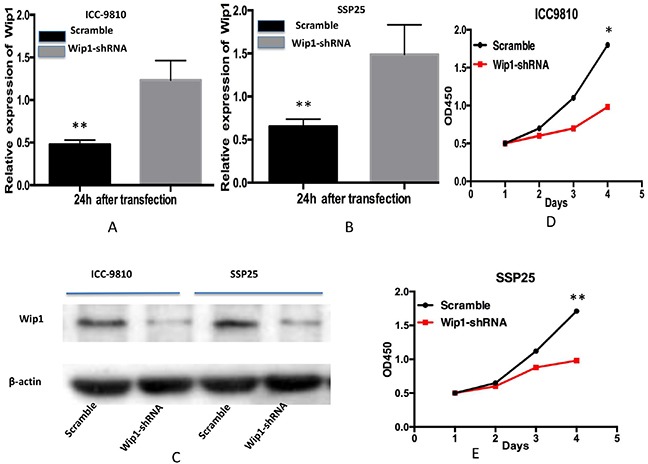
Transfection of Wip1-shRNA represses proliferation of ICC-9810 and SSP25 cells *in vitro* Real time PCR analysis revealed that Wip1 mRNA in Wip1-shRNA ICC-9810 and SSP25 cells was significantly down- regulated **(A,B)** Similar results were obtained by Western blotting analysis **(C)** Down-regulation of Wip1 expression by Wip1-shRNA significantly inhibited the proliferation of ICC-9810 and SSP25 cells cells **(D,E)** *P<0.05, ** P<0.001.

**Figure 3:**
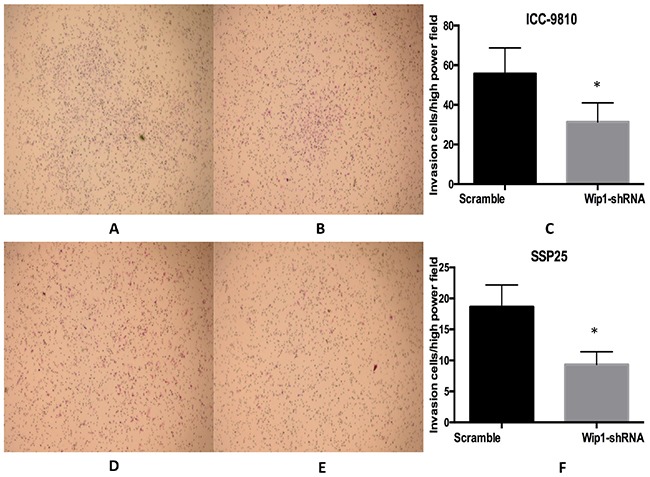
Wip1 knockdown inhibited the invasive ability of ICC cells *in vitro* Representative images of the invaded ICC-9810 and SSP25 cells in Matrigel invasion assays were taken at ×200 magnification **(A,B,D,E)** Numbers of the invaded ICC-9810 and SSP25 cells per microscopic field **(C,F).** * p<0.05.

We then determined the effect of Wip1 on cell proliferation and invasion in two ICC cell lines. We depleted its expression in ICC-9810 and SSP25 cells by shRNA-Wip1 and established stable cell lines. As shown in Figure 2, the qRT-PCR and Western blot results revealed Wip1 was effectively and functionally suppressed in ICC-9810 and SSP25 cells compared to the scramble control (Figure 2A–2C). To further explore the biological role of reduced Wip1 of ICC-9810 and SSP25 cell lines, proliferation assay was performed. CCK-8 cell proliferation assay showed that the decrease in Wip1 expression caused by Wip1-shRNA obviously inhibited the proliferation of ICC-9810 and SSP25 cells (Figure 2D,2E). Next, we assessed the alterations to the capacity of invasion in ICC-9810 and SSP25 cells lines. As show in Figure 3A–3C, Madrigal invasion assay revealed that the decrease in Wip1 expression caused by Wip1-shRNA significantly inhibited ICC-9810 cells’ invasion capacity. Similar results were also obtained in SSP25 cells (Figure 3D–3F). These data vividly indicated that down- regulation of Wip1 inhibits the proliferation and invasiveness of ICC-9810 and SSP25 cells.

### Transfection of Wip1-shRNA impaired the migration of ICC-9810 and SSP25 cells *in vitro*

**Figure 4:**
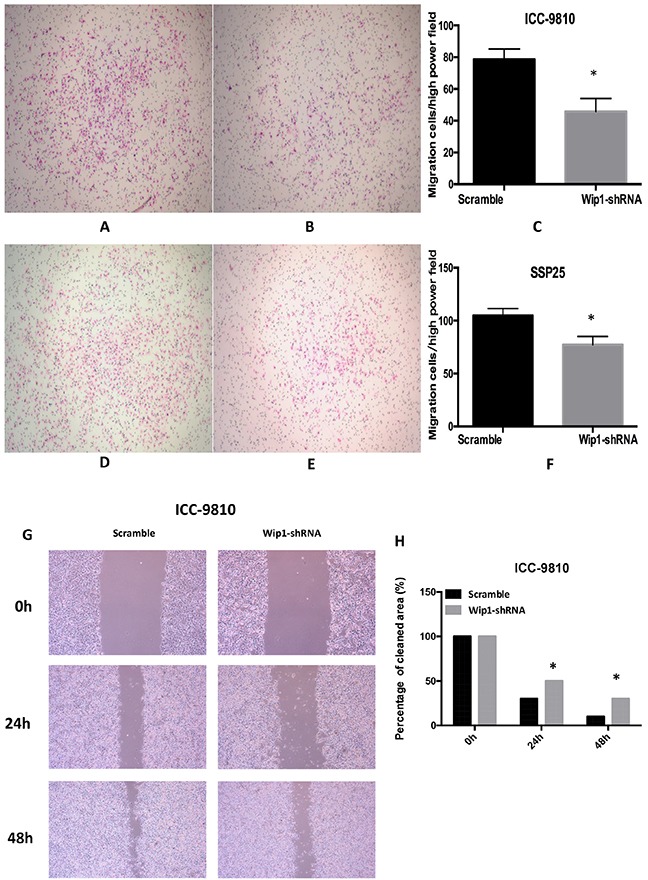
Wip1 knockdown inhibited the migration ability of ICC cells *in vitro* Representative images of the migrate ICC-9810 and SSP25 cells in Matrigel assays were taken at ×200 magnification **(A,B,D,E)** Numbers of the invaded ICC-9810 and SSP25 cells per microscopic field **(C,F).** In a wound healing assay, ICC-9810 and SSP25 cells in the Wip1-shRNA group exhibited decreased migration ability compared with control group **(G)** Cells were monitored every 24 h for 2 days to evaluate the rate of migration into the scratched area (H).*P<0.05.

To examine whether the targeted down-regulation of Wip1 in ICC-9810 and SSP25 cells affects the migration of tumor cells, migration assays were performed. Our results show that cells in the Wip1-shRNA in ICC-9810 group exhibited decreased migration ability compared with the Scramble (Figure 4A–4C). Similar results were found in SSP25 cells tranfected with Wip1-shRNA (Figure 4D–4F). Thus, down-regulation of Wip1 dramatically diminished the migration of ICC-9810 and SSP25 cells *in vitro*. Consistently, wound healing assays show that cells in the Wip1-shRNA group exhibited decreased migration ability compared with the control (Figure 4G–4H). Similar results were observed in SSP25 cell line. Therefore, down-regulation of Wip1 dramatically decreased the migration of ICC-9810 and SSP25 cells *in vitro*.

### Knockdown of Wip1 repressed MMP-2 expression in ICC-9810 and SSP25 cells

**Figure 5:**
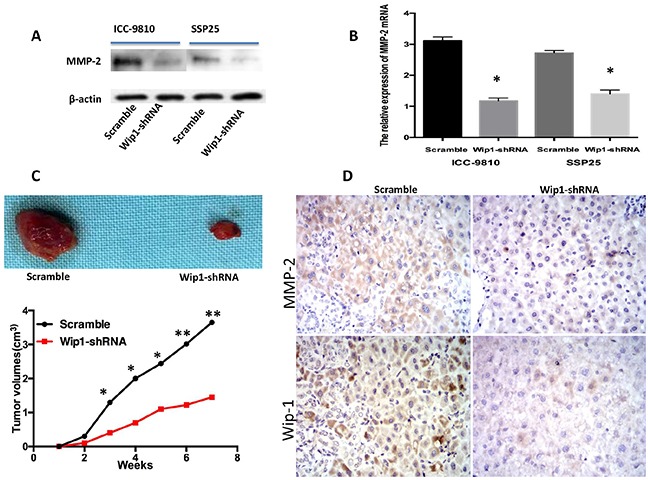
Transfection of Wip1-shRNA impaired ICC-9810 and SSP25 cell tumor formation through MMP-2 *in vivo* Western blot/PCR revealed that shRNA-Wip1 remarkably decreased the levels of MMp-2 **(A,B)** Following down- regulation of Wip1 expression, ICC-9810 and SSP25 cells exhibited significantly diminished *in vivo* tumor formation ability compared with control cells (**C**, upper). Tumor size was measured every week. There was a significant reduction in relative tumor volume from shRNA-Wip1-treated animals when compared with untreated controls (**C**, down). The expression of Wip1/MMP-2 was positively correlated with subcutaneous tumor volume **(D).** *P<0.05.

We next investigated a potential mechanism for Wip1-mediated ICC cell migration and invasion. Typically, malignant cells could breakage of the basement membrane through secreting Matrix metalloproteinases (MMPs) that digest the extracellular matrix (ECM) proteins. Accumulating studies have revealed that MMP-2 was an important regulator of metastasis in ICC [20, 21]. Indeed, we have recently found new candidates Wip1 substrate (contain MMP-2), which localizes in the cytoplasm and suppresses cell death signaling linked to ICC (manuscript in preparation). We therefore investigated MMP-2 expression using immunohistochemistry on tissues from all 60 patients with ICC. MMP-2 levels were positively associated with Wip1 expression (Figure 1E–1H and Table 1; P=0.039). Further more, the expression of Wip1 and MMP-2 in ICC were correlated with hilar lymph nodes metastasis. To further identify the effect of Wip1 expression in ICC cell lines *in vitro*, our data show the introduction of shRNA-Wip1 remarkably decreased MMP-2 protein expression in ICC-9810 and SSP25 cells (Figure 5A). In accordance with this, the MMP-2 mRNA level was observed significantly lower in transfect Wip1-shRNA cells than Scramble cells (P<0.05; Figure 5B).

Finally, we established xenograft-using cells treated respectively with ICC-9810 Wip1-shRNA and control cells, and documented the tumor volume weekly. Our data show there was no difference of tumor volume between the ICC-9810 Wip1-shRNA and control groups in the first two weeks (Figure 5C), but the growth of the tumor in the Wip1-shRNA group significantly slowed down since the 3th week, compared with the control group (P<0.05). Wip1 and MMP-2 expression in subcutaneous tumors were analyzed by immunohistochemistry. Wip1 and MMP-2 immunohistochemical stainings in the Wip1-shRNA tumors were remarkably lower than that in control, indicating that interference for Wip1 may restrain the proliferation of tumor cells through MMP-2 (Figure 5D).

## DISCUSSION

Wip1, on chromosome 17q22-q23, functions as an oncogene and inhibits p53 activity when overexpressed in proliferation of certain self- renewing cell types, including carcinogens, with advancing age [22]. These features were mainly connected with Wip1 ability to regulate signaling in DNA damage response (DDR) and MAPK kinases pathway p53 network, including p38, p53, ATM, Chk2, and γ-H2AX [7, 23, 24]. Accumulating studies showed that over expression of Wip1 will disrupt multiple pathways implicated in the regulation of p38MAPK-p53 tumor suppressor responses, which caused down target Wnt-p53 dephosphrylation through limiting the p38MAPK-STAT1 pathway, and promoted tumor formation in humans by decreasing p16/p19 levels [11, 25]. Our previous data have indicated that Wip1 was an oncoprotein and was involved in controlling mechanism in renal carcinoma cells [14]. Sona et al report that Inhibition of Wip1 by GSK2830371 sensitizes breast cancer cells to senescence and to activation of caspase-9 [25]. A recent research article demonstrated that crosstalk among Wip1, CXCR4 and GRK5 promote aggressive phenotype of a medulloblastoma in children [9]. However, the role of Wip1 in the tumorigenicity and metastasis of ICC cells was scarcely investigated. Here, we show that the expression of Wip1 was elevated in ICC tissues, and the over expression of Wip1 was associated with lymph node metastasis. These data indicated that Wip1 may involve in the lymph node metastasis of ICC.

ICC is a fatal primary liver carcinoma, which originating from the intrahepatic bile duct epithelium beyond the second-order bile ducts [5] [26, 27]. ICC is the second most common all primary liver cancers in the worldwide, the actual incidence has been increasing steadily and substantially over the past decades and has a poor outcomes and high mortality rate due to the high invasiveness and recurrence [1, 28–30]. Some previous studies reported that Wip1 expression was associated with miR-17-5p through activation of the p38 pathway in hepatocellular carcinoma (HCC)[31]. Zhang L et al show that inhibition of Wip1 can activate the mammalian target of rapamycin complex 1 (mTORC1) pathway and enhance hepatocyte proliferation after hepatectomy [32]. However, in line with another study on Asian patients with HCC [33, 34], Our data found Wip1 expression in up to 78.3% (47/60) of ICC cases, while in none of adjacent cancer tissues, suggesting that ICC and HCC histological appearance were very similar and a significant racial difference.

In this study, our data show that Wip1 was over-expressed in human ICC cases, which was significantly correlated with aggressive phenotypes of cancer cells. Furthermore, our results found that depletion of Wip1 inhibited the proliferation and migration of human ICC cells. Recent reports have indicated that Wip1-knockout mice impaired their tumor formation capacity *in vivo*. What's more, even when tumors form in these kinds nude mice, the carcinoma cells often show a low proliferation potential [35]. Our *in vivo* dada show that Wip1 down- expression in nude mice inhibits tumor formation capacity and decreases cancer progression.

In this study, we indicated that the Wip1 expression in ICC was significantly associated with lymph metastasis and nerve invasion (p<0.001, p=0.035 respectively). Moreover, our data show that the wip1 was significantly correlated with P53 (p=0.014), CA-199 (p=0.015) and MMP-2 (p=0.008) expression. A body of evidence indicated that Wip1 was a P53-regulated oncogene, which inactivated downstream proteins including P38, P53, ATM, Chk2 and turn back the tumor cells to a homeostasis state when induced by environment stresses [11, 24, 32]. Here, we have indicated depletion of Wip1 show impaired proliferation and migration capacity in ICC cells. What's more, implantation of Wip1-shRNA ICC cell into mice inhibited tumorigenesis. This suggests down-expression of Wip1 inhibits ICC migration and invasion.

We next study the mechanism of wip1 in the oncogenesis and metastasis of ICC. In the present study, up-regulations of MMP-2 and CA199 are the markers for increased proliferation of cancer cells. Further results showed that Wip1 knock-down by Wip1-shRNA dramatically inhibited the invasion and migration of ICC-9810 and SSP25 cells and decreased MMP-2 expression. MMP-2, as a key protein in the ECM and basement membrane, that can promote carcinoma cell invasion and metastasis [36, 37].

A body of evidence indicated that lymph node metastasis was one of the strongest prognostic factors associated with survival in ICC patients [38–40]. Most studies reported that lymph node metastasis has a powerful negative effect on survival in these patients [3, 38]. In this study, Wip1 expression levels were dramatic associated with increased lymph node metastasis. These data show that Wip1 promoted lymphatic metastasis in ICC patients. Furthermore, We demonstrated that Wip1-shRNA significantly inhibited MMP-2 expression in ICC cells, which was in collaborate with a previous report that knock-down the expression Wip1 can inhibit migration and invasion target VEGF-C and MMP-9 pathway in salivary adenoid cystic carcinoma [41].

At the histological level, ICC is characterized by an abundant stroma; i.e. the tumor microenvironment that notably includes components of the ECM, stromal cells and soluble factors [42]. On the other hand, MMPs (Matrix metalloproteinases) have an crucial effect in tumor cell invasion and metastasis by degrading ECM proteins [43]. MMP-2 is a secreted protein implicated in the destruction of broad range of ECM substrates in various cancers [44]. In this study, Wip1 knockdown inhibited ICC cell motility and invasion, and decreased the expression of EMT markers MMP-2. The regulation of MMP-2 expression is via various transcription factors such as nuclear factor κB (NFκB), mitogen-activated protein kinases (MAPKs), and phosphoinositide-3 kinase/protein kinase B (PI3K/AKT) pathways [44, 45]. Based on above data, we suggest that the high level of Wip1 may induce ICC cells invasion and metastasis by up-regulating the MMP-2 expression.

In conclusion, our study demonstrate that wip1 expression is a crucial regulator in the progression of human ICC; further investigations show Wip1 exerts its pro-invasion function at least in part through the MMP-2 signaling pathway, suggesting that Wip1 may be a potential target for ICC therapy.

## MATERIALS AND METHODS

### Patients and tissue source

Sixty primary ICC and distant normal tissue samples were obtained from ICC patients that underwent radical surgical resection between June 2014 and July 2016 at The Department of Hepatobiliary Surgery, Hunan Provincial People's Hospital/The First Affiliated Hospital of Hunan Normal University. None of the patients received any preoperative chemo- and radiotherapy, or other medical interventions. The clinicopathological characteristics of the patients were retrieved from the medical records and are summarized in Table 1. The fresh specimens of tumor tissue or adjacent normal epithelium 1 cm apart from the tumor edge were immediately taken after the surgery, which was fixed in 4% paraformaldehyde solution, and then embedded in paraffin for immunohistochemistry. This study was reviewed and approved by the Ethics Committee of The Hunan Normal University (NO:566789), and an informed consent form was signed by each patient before surgery. All methods were carried out in accordance with relevant guidelines and regulations of The Hunan Normal University (Two edition 2013).

### Evaluation of immunohistochemical staining

The immunohistochemical staining of Wip1 on ICC tissue was evaluated in accordance with our previous studies [14, 19]. Briefly, all tissue sections were reviewed under a light microscopy and scored for at least five fields at a×400 magnification independently by two pathologists who were unaware of any clinical or outcome data.

The scoring system was as follows: the percentage of positively staining cells was graded as - (no staining, Wip1-negative), + (>0 and ≤ 25% of cells positive, weak Wip1-positive), ++ (>25 and ≤75% of cells positive, moderate Wip1-positive), and +++ (>75% of cells positive, strong Wip1-positive).

### Reagents and antibodies

DMEM, lipofectamine 2000, trypsin, trizol reagent, penicillin/streptomycin and fetal bovine serum (FBS) were from Invitrogen. The rabbit polyclonal anti-Wip1 and rabbit polyclonal anti-MMP-2 (Cell Signaling Technology, Beverly, MA) was used at the dilution of 1:400, while β-actin (Santa Cruz Biotechnology, Santa Cruz, CA) was used as a loading control. In accordance with Wip1 gene sequence in the NCBI database and siRNA design principles, the Wip1-specific shRNAs was designed and synthesized by Shanghai ShengGong Co., Ltd., DNA oligonucleotides used to generate shRNAs against the open reading frame of mRNA were 5′-CCCTTCTCGTGTTTGCTTAAA-3′ (for Wip1) to silence the expression of Wip1. In addition, a negative shRNA control that shared no homology to siRNA-Wip1 genome sequence was designed and synthesized by Shanghai ShengGong Co. Ltd.

### Cell lines and culture

ICC-9810 and SSP25 were purchased from the Type Culture Collection of the Chinese Academy of Sciences (Shanghai, China); BEC (humanbiliary epithelial cell) were preserved in our laboratory. Cells were grown in DMEM supplemented with 10% FBS and penicillin or rstreptomycin (100 U/mL/50mg/mL) at 5% CO2 and 37°C.

### Cell proliferation, invasion, migration, western blot and quantitative real-time PCR assays

Cell proliferation, invasion, migration assay were carried out as our previously described [14]. Total protein was extracted using RIPA buffer and protein expression was analyzed by western blot as our described study [14]. Total RNA was extracted and purified from cell lines according to the manufacturer's instructions and our previous report [14].β-actin served as an endogenous control. The sequences of gene specific primers for Wip1 (forward, 5′- GAAGGATGACTTTGTCAG -3′; reverse, 5′- CCCAGACTTGTTCATTAC-3′) andβ-actin (forward, 5′- CATCCTGCGTCTGGACCTGG -3′; reverse, 5′- TAATGTCACGCACGATTTCC -3′) were designed using NCBI Primer-BLAST.

### Wound healing assay

Wound healing assay was performed to evaluate the cell migratory capacity. In brief, ICC-9810/SSP25 cells were cultured to full confluence. Wounds of approximately 1 mm width were created with a plastic scriber, and cells were washed. After cultured for 24/48 hours, cells were fixed and observed under a microscope.

### Xenografts in nude mice

All animal experiments described in this study was performed in accordance with protocols approved by the Institutional Animal Care and Use Committee at Hunan Normal University. Briery, 5 × 10^6^ shRNA-Wip1 ICC cells and control cells were suspended in 100 μl PBS and were injected subcutaneously into six female nude mice (Balb/c nu/nu) (3-4 weeks old), respectively. Tumor volumes were monitored every 7 days by measuring the length and width with a caliper and using the formula (width2) × length/2. Mice were sacrificed 7 weeks after injection, and the tumors were isolated and measured.

### Statistical analysis

Statistical analyses were performed using GraphPad Prism6 software (Graphpad Software, Inc., La Jolla, CA, USA). Values were expressed as mean±SD using the Student's t-test. The correlation analysis was examined using spearman rank correlation test. Data are presented as the means ± SD from three independent experiments. The p<0.05 was considered statistically significant.
